# Neurogenetic Profiles of Anxiety, Impulsivity, and Personality Traits in Elite Combat Sport Athletes: A Cluster-Based Analysis

**DOI:** 10.3390/biology15030290

**Published:** 2026-02-06

**Authors:** Kinga Humińska-Lisowska, Remigiusz Recław, Aleksandra Suchanecka, Krzysztof Chmielowiec, Kinga Łosińska, Jolanta Chmielowiec, Anna Grzywacz

**Affiliations:** 1 Department of Molecular Biology, Gdansk University of Physical Education and Sport, Kazimierza Górskiego 1 St., 80-336 Gdansk, Poland; kinga.huminska-lisowska@awf.gda.pl; 2 Independent Laboratory of Genetics and Behavioral Epigenetics, Pomeranian Medical University in Szczecin, Powstańców Wielkopolskich 72 St., 70-111 Szczecin, Poland; remigiusz.reclaw@pum.edu.pl (R.R.); aleksandra.suchanecka@pum.edu.pl (A.S.); 3 Department of Medical Sciences and Public Health, Gdansk University of Physical Education and Sport, Kazimierza Górskiego 1 St., 80-336 Gdansk, Poland; kinga.losinska@awf.gda.pl; 4 Department of Hygiene and Epidemiology, Collegium Medicum, University of Zielona Góra, 28 Zyty St., 65-046 Zielona Góra, Poland; chmiele@vp.pl; 5 Department of Nursing, Collegium Medicum, University of Zielona Góra, 28 Zyty St., 65-046 Zielona Góra, Poland; j.chmielowiec@inz.uz.zgora.pl

**Keywords:** neurogenetics of behaviour, dopamine signaling, *DRD2*, *DRD4*, *DAT1*, personality traits, anxiety, impulsivity, cluster analysis, elite athletes

## Abstract

Behavioral regulation in elite combat sports relies on traits such as anxiety, impulsivity, and personality, which are partly shaped by dopaminergic signaling. However, integrative approaches linking multidimensional behavioral profiles with genetic variability in athletes remain limited. This study aimed to identify distinct psychological profiles in elite combat athletes and examine whether these profiles differ in selected dopaminergic gene polymorphisms. A total of 200 male Polish elite combat athletes completed validated questionnaires assessing personality, anxiety, impulsivity, attention-related symptoms, and hedonic capacity. Standardized psychological variables were analyzed using a two-step clustering procedure. Genotype distributions were compared across clusters using chi-square tests. The clusters showed clear behavioral differentiation. Cluster 1 demonstrated higher anxiety, impulsivity, neuroticism, and attention-related symptoms. Cluster 2 showed lower anxiety and impulsivity alongside higher extraversion and conscientiousness. Cluster 3 displayed intermediate behavioral characteristics but the most distinctive genotype pattern. These findings suggest that combining behavioral clustering with genetic data may help characterize meaningful neurogenetic profiles in elite athletes and provide hypothesis-generating insights into genotype–phenotype relationships.

## Simple Summary

Elite combat sport athletes must control stress, attention, and impulsive reactions during training and competition. However, athletes differ markedly in these psychological characteristics, and the biological factors behind these differences are still not well understood. In this study, we examined whether athletes could be grouped into distinct psychological profiles and whether these profiles were linked to differences in selected dopamine-related genetic variants. We assessed 200 male elite Polish combat athletes using questionnaires measuring personality, anxiety, impulsivity, attention-related symptoms, and the ability to experience pleasure. Using statistical grouping methods, we identified three different athlete profiles. One group showed higher anxiety, higher impulsivity, and more attention-related symptoms. Another group showed lower anxiety and impulsivity with higher extraversion and conscientiousness. A third group showed intermediate behavioral levels but was the most genetically distinctive. These findings suggest that combining psychological profiling with genetic information may help describe meaningful subtypes of athletes. In the future, this approach could support more individualized psychological monitoring and training strategies in high-performance sport.

## 1. Introduction

Behavior represents a complex and multidimensional phenotype shaped by dynamic interactions between genetic predisposition and environmental influences [1,2,3]. Increasing evidence indicates that inter-individual variability in behavioral traits cannot be adequately explained by single genes or isolated psychological dimensions, but rather emerges from the combined effects of multiple genetic variants and their influence on neurobiological systems [4]. In this context, neurogenetics of behaviour provides an important framework for understanding how genetic variation contributes to stable personality traits, emotional regulation, and individual differences in behavioral responses across populations [5,6]. However, despite substantial progress in identifying candidate genes associated with behavior, the translation of genetic variability into coherent behavioral profiles remains a major challenge in contemporary research [7].

Among the neurobiological systems implicated in the regulation of behavior, the dopaminergic system plays a central role in shaping emotional reactivity, motivational processes, reward sensitivity, and behavioral control [8]. Dopamine signaling has been consistently linked to key behavioral dimensions such as anxiety, impulsivity, novelty seeking, and attentional regulation, which together form core components of individual behavioral profiles [9]. Genetic variation within dopaminergic pathway genes, including dopamine receptors, transporters, and related regulatory proteins, has been shown to contribute to inter-individual differences in these traits [10,11]. Consequently, polymorphisms within dopaminergic genes have become a major focus of neurogenetic research aimed at elucidating biological substrates underlying variability in behavior [12].

Anxiety, impulsivity, attentional regulation, and stable personality traits represent closely interconnected dimensions of behavior that are frequently examined independently, despite substantial evidence for their overlap at both behavioral and neurobiological levels [13]. Elevated anxiety is often accompanied by increased impulsivity and attentional difficulties, while personality traits such as neuroticism and conscientiousness modulate emotional reactivity and behavioral control [14,15]. These traits are not discrete entities but form interacting constellations that shape individual behavioral functioning [16]. From a neurogenetic perspective, this multidimensionality suggests that behavioral variability is more accurately captured by integrated profiles rather than by isolated trait measures, highlighting the need for analytical approaches that consider the co-occurrence and interaction of multiple behavioral dimensions [17].

Traditional analytical approaches in behavioral genetics often rely on examining associations between individual genetic variants and single behavioral traits, which may overlook the inherent heterogeneity of behavioral phenotypes [18,19]. Such approaches assume relative homogeneity within study populations and fail to account for the complex, multidimensional structure of behavioral regulation [20]. Unsupervised clustering methods offer an alternative framework by enabling the identification of naturally occurring subgroups characterized by distinct constellations of behavioral features [21]. In particular, cluster-based analyses allow for the integration of multiple psychological dimensions into coherent profiles, providing a data-driven approach to capturing behavioral diversity without imposing predefined assumptions regarding trait boundaries [22].

In contrast to correlation or regression models, which typically estimate average (often linear) associations between single genetic variants and individual behavioral traits at the sample level, cluster-based approaches are designed to capture latent heterogeneity and multivariate patterns of trait co-occurrence. In the context of behavioral genetics, genetic effects may manifest not as isolated shifts in a single dimension but as distinct constellations of anxiety, impulsivity, attentional regulation, and personality features that form naturally occurring subgroups. Therefore, an unsupervised clustering framework may provide complementary insights by identifying empirically derived behavioral profiles that would remain obscured when traits are analyzed separately.

Elite combat sport athletes constitute a unique and informative model for investigating neurogenetic mechanisms underlying behavioral regulation [23,24]. This population is characterized by prolonged exposure to high cognitive, emotional, and physical demands, requiring precise control of anxiety, impulsivity, attention, and motivational processes [25]. At the same time, elite athletes represent a highly selected group in which behavioral traits are shaped by both genetic predispositions and long-term adaptive processes [26]. From a neurogenetic perspective, studying such populations provides an opportunity to examine how genetic variability contributes to differentiated behavioral profiles under conditions of sustained performance-related pressure, offering insights that may not be readily observable in more heterogeneous general populations [27].

Despite growing evidence linking dopaminergic genetic variation to individual behavioral traits, studies integrating multiple neurobehavioral dimensions with genetic profiles using data-driven approaches remain limited. Most existing studies have examined dopaminergic polymorphisms in relation to single behavioral outcomes, which limits the ability to describe multidimensional behavioral phenotypes as integrated profiles [26,28]. Moreover, evidence for data-driven neurobehavioral clustering combined with genetic profiling remains scarce, particularly in highly selected high-performance populations such as elite combat sport athletes. To our knowledge, this is one of the first studies to apply an unsupervised clustering strategy integrating anxiety, impulsivity, attentional regulation, and personality traits, and to subsequently examine the distribution of selected dopaminergic polymorphisms across the resulting behavioral profiles in elite combat sport athletes.

Therefore, the aim of the present study was to investigate whether elite combat sport athletes can be differentiated into distinct clusters based on integrated measures of anxiety, impulsivity, attentional regulation, and personality traits, and to examine the distribution of selected dopaminergic gene polymorphisms across these clusters. Identifying such behavioral profiles may improve the interpretability of neurogenetic variability in elite sport settings and provide a foundation for more individualized profiling and psychological monitoring. By applying this data-driven approach, the present study seeks to provide an integrative perspective on neurogenetic variability underlying behavioral profiles in elite athletes [29]. Accordingly, behavioral clusters were first derived based on the joint distribution of multiple psychological measures, and the prevalence of dopaminergic genetic variants was subsequently compared across the identified profiles.

## 2. Materials and Methods

### 2.1. Participants

The study included 200 male Polish combat sport athletes with a mean age of 22.86 ± 6.25 years. Participants represented five combat sport disciplines: judo (*n* = 51), wrestling (*n* = 38), boxing (*n* = 50), kickboxing (*n* = 32), and karate (*n* = 29). All athletes were active competitors and ranked among the top ten athletes at the national level in their respective disciplines at the time of recruitment. Exclusion criteria included a history of traumatic brain injury and current clinically relevant psychiatric symptoms or disorders (e.g., major depressive episodes, psychotic symptoms, or substance dependence), assessed using the Mini-International Neuropsychiatric Interview (MINI). Data on training volume, recent competition exposure, and acute psychological stress at the time of assessment were not systematically collected and were therefore not included as covariates in the statistical analyses.

Based on competitive achievements, athletes were categorized into three performance levels. Sixteen participants were classified as top-elite, defined as gold medalists at World Championships, European Championships, World Cups, or Olympic Games. Sixty-eight athletes were classified as elite, having obtained silver or bronze medals at major international competitions. The remaining participants (*n* = 116) were classified as sub-elite, defined as athletes with at least eight years of competitive experience who had participated in international competitions without achieving podium finishes.

Participants were recruited through collaboration with national sport federations, national team coaching staff, and during centralized training camps. All individuals were free from self-reported psychiatric disorders, including psychotic disorders and substance dependence, at the time of participation, and participants reporting current psychiatric disorders were not eligible for inclusion.

### 2.2. Measures

Personality traits, anxiety, impulsivity, attention-deficit/hyperactivity symptoms, and hedonic capacity were assessed using a battery of standardized and widely validated self-report questionnaires.

Personality Traits (NEO-FFI). Personality dimensions were evaluated using the NEO Five-Factor Personality Inventory (NEO-FFI), which assesses five core domains of personality: Neuroticism, Extraversion, Openness to Experience, Agreeableness, and Conscientiousness. The NEO-FFI is based on the five-factor model of personality and is extensively used in both clinical and non-clinical research settings. Participants responded to items using a five-point Likert-type scale, and raw scores were converted into standardized sten scores in accordance with normative data. Higher scores reflect greater expression of the respective personality trait.

Anxiety (STAI). Anxiety levels were measured using the State–Trait Anxiety Inventory (STAI), which distinguishes between anxiety as a transient emotional state (state anxiety) and anxiety as a stable personality characteristic (trait anxiety). The questionnaire consists of two separate 20-item subscales, each rated on a four-point Likert scale. Raw scores were converted into standardized sten scores, which were used in all subsequent statistical analyses, with higher values indicating greater anxiety severity. The STAI has demonstrated high reliability and validity in diverse populations, including athletes and clinical samples.

Impulsivity (BIS-11). Impulsivity was assessed using the Barratt Impulsiveness Scale, version 11 (BIS-11), a 30-item self-report questionnaire measuring three dimensions of impulsivity: Attentional Impulsivity (AI), Motor Impulsivity (MI), and Non-Planning Impulsivity (NI). Participants rated each item on a four-point Likert scale, with higher scores indicating greater impulsivity. In addition to subscale scores, a total impulsivity score was calculated. The BIS-11 is a well-established instrument with strong psychometric properties and is frequently applied in studies examining behavioral regulation, addiction-related traits, and dopaminergic functioning.

ADHD Symptoms (ASRS v1.1). Symptoms associated with attention-deficit/hyperactivity disorder were evaluated using the Adult ADHD Self-Report Scale (ASRS v1.1). This 18-item questionnaire, developed by the World Health Organization, assesses core ADHD symptoms related to inattention and hyperactivity/impulsivity in adults. Responses are provided on a five-point Likert scale ranging from “never” to “very often,” with higher total scores indicating greater symptom severity. The ASRS v1.1 is widely used as a screening tool and demonstrates good reliability in adult populations.

Hedonic Capacity (SHAPS). Hedonic capacity was measured using the Snaith–Hamilton Pleasure Scale (SHAPS), a 14-item self-report instrument designed to assess the ability to experience pleasure across various domains of daily life, including social interaction, sensory experience, and personal interests. Items are rated on a four-point scale, with higher scores reflecting greater pleasure sensitivity and lower levels of anhedonia. The SHAPS has been validated in both clinical and non-clinical samples and is considered a reliable measure of hedonic functioning.

### 2.3. Genotyping

Peripheral blood samples were collected from all participants, and genomic DNA was extracted using commercially available isolation kits following standardized laboratory protocols (Roche, Basel, Switzerland). Genotyping analyses were conducted using molecular methods appropriate for the type of genetic variation investigated, including single-nucleotide polymorphisms (SNPs) and variable number tandem repeat (VNTR) polymorphisms.

Single-nucleotide polymorphisms in the *DRD2* and *ANKK1* genes were genotyped using real-time polymerase chain reaction combined with allele-specific fluorescent probes and melting curve analysis. Analyses were performed with LightSNiP assays (TIB MOLBIOL, Berlin, Germany) on a LightCycler 480 platform (Roche Diagnostics GmbH, Mannheim, Germany). Each run included negative (no-template) controls, and a randomly selected subset of samples was reanalyzed to confirm genotyping accuracy.

Allele discrimination was based on characteristic melting temperatures. For *DRD2* rs1076560, melting peaks were observed at approximately 57.4 °C for the A allele and 64.4 °C for the C allele. The *DRD2* Tag1D rs1800498 polymorphism showed melting peaks at approximately 56.6 °C (C allele) and 62.9 °C (T allele), whereas *DRD2* Tag1B rs1079597 was identified by peaks at approximately 57.4 °C (G allele) and 62.3 °C (A allele). For *DRD2* Ex8 rs6276, allele-specific melting peaks were detected at approximately 59.1 °C for the G allele and 67.7 °C for the A allele. The *DRD2* promoter polymorphism rs1799732 was distinguished by melting peaks at approximately 56.5 °C for the insertion allele and 62.9 °C for the deletion allele. For the *ANKK1* Tag1A rs1800497 polymorphism, characteristic melting peaks were observed at approximately 67.2 °C for the C allele and 59.0 °C for the T allele.

Genotyping of VNTR polymorphisms was performed using fragment length analysis following polymerase chain reaction amplification. For the *DAT1* gene, the VNTR located in the 3′ untranslated region was classified based on fragment sizes corresponding to the 9-repeat and 10-repeat alleles. The *DRD4* exon 3 VNTR polymorphism was categorized into short (s) and long (l) alleles according to PCR product length. These allele classifications were used in all subsequent statistical analyses.

### 2.4. Statistics

Descriptive statistics for psychological variables were calculated using the mean (*M*), standard deviation (*SD*), 95% confidence intervals for the mean (95% CI), median (*Mdn*), and range, as appropriate. Genotype distributions for the analyzed polymorphisms were summarized as absolute frequencies and percentages. A Pearson’s correlation heatmap was used to illustrate relationships between the assessed psychological dimensions and to detect potential multicollinearity.

A two-step clustering procedure was applied. First, hierarchical cluster analysis was conducted as an exploratory step to determine the most plausible number of clusters. Visual inspection of the dendrogram and the agglomeration schedule indicated a clear increase in linkage distance when moving from three to two clusters, which supported a three-cluster solution (k = 3) in accordance with an elbow-type criterion. In the second step, k-means clustering was performed using Euclidean distance in STATISTICA, with a maximum of 50 iterations and an initialization procedure aimed at maximizing the distance between cluster centroids. Cluster stability was assessed indirectly by examining the reproducibility of the cluster structure across successive iterations of the algorithm.

Following cluster assignment, differences between clusters in quantitative variables were examined using one-way analysis of variance (ANOVA). Effect sizes were reported as η^2^ (eta squared), indicating the proportion of variance explained by cluster membership. Differences in genotype frequencies across clusters were assessed using the chi-square (χ^2^) test of independence. To control the type I error for the STAI, NEO-FFI, BIS-11, ADHD, and SHAPS tests, a Bonferroni correction was applied, adjusting the significance level to α = 0.0038 (0.05/13). To control the type I error for comparisons of analyzed gene polymorphisms, the Bonferroni correction was applied, adjusting the significance level to α = 0.00625 (0.05/8). All tests were two-tailed, and statistical significance was interpreted at *p* < 0.05 unless otherwise specified by Bonferroni-adjusted thresholds.

Statistical analyses were conducted using STATISTICA version 13 (TIBCO Software Inc., Palo Alto, CA, USA) and PQStat version 1.8.6 (PQStat Software, Poznań, Poland) for Windows (Microsoft Corporation, Redmond, WA, USA).

## 3. Results

The mean age of the participants was 22.86 years (95% CI: 21.99–23.73), with a median of 21 years and standard deviation (*SD* = 6.25). Both trait anxiety (STAI trait) and state anxiety (STAI state) showed comparable mean levels (*M* = 5.28 and *M* = 4.90, respectively), with medians of 5, indicating a moderate level of anxiety in the study sample.

Analysis of personality traits revealed the highest mean scores for conscientiousness (*M* = 7.18) and extraversion (*M* = 6.20), whereas lower mean values were observed for openness to experience (*M* = 4.42) and neuroticism (*M* = 4.84). Median values for all personality dimensions were close to the corresponding means, suggesting approximately symmetric score distributions.

Regarding impulsivity assessed with the BIS-11, the mean total score was 61.97 (95% CI: 60.75–63.19). Among the BIS-11 subscales, non-planning impulsivity (BIS-NI) showed the highest mean value, whereas attentional impulsivity (BIS-AI) exhibited the lowest.

The mean level of ADHD symptoms was 22.48, with relatively high variability (*SD* = 11.53), indicating substantial heterogeneity within the study group. In contrast, scores on the SHAPS, reflecting hedonic capacity, demonstrated moderate variability (*M* = 46.43; *SD* = 8.19) (Table 1).

To examine correlations between the assessed psychological traits and to identify potential multicollinearity, a Pearson correlation heatmap was generated (Table 2). This visualization allowed for a clear overview of the strength and direction of relationships among the variables. Pearson correlation coefficients above |r| = 0.8 were observed only for STAI state vs. STAI trait and for STAI trait vs. neuroticism. This suggests that strong multicollinearity was limited to these pairs.

For the *DRD2* rs1076560 polymorphism, the C/C genotype was the most prevalent, occurring in 80% of participants, whereas the heterozygous A/C genotype accounted for 16% and the A/A genotype for 4% of the sample. A similar distribution was observed for *DRD2* Tag1B rs1079597, with a predominance of the G/G genotype (86%) and no individuals carrying the A/A genotype.

In the case of *DRD2* Tag1D rs1800498, the T/T genotype was the most frequent (60%), while the C/T and C/C genotypes were observed in 28% and 12% of participants, respectively. A more heterogeneous genotype distribution was noted for *DRD2* Ex8 rs6276, where the A/A genotype occurred in 42% of the sample, followed by A/G (38%) and G/G (20%).

Regarding the *DRD2* promoter polymorphism rs1799732, the ins/ins genotype predominated (74%), whereas the ins/del genotype was identified in 26% of participants; no individuals with the del/del genotype were observed. Similarly, for the *ANKK1* Tag1A rs1800497 polymorphism, most participants were homozygous C/C (80%), with the remaining 20% being heterozygous C/T and no T/T genotype detected.

Analysis of VNTR polymorphisms showed that for *DAT1*, the 10/10 and 9/10 genotypes were equally frequent, each occurring in 48% of participants, while the 9/9 genotype was rare (4%). For *DRD4* Ex3, the s/s genotype was the most common (58%), followed by s/l (40%), whereas the l/l genotype was infrequent (2%) (Table 3).

Following the analysis of genotype distributions (Table 3), cluster analysis was performed in the athletic cohort comprising 200 participants using a two-step approach. First, hierarchical cluster analysis supported a three-cluster solution (k = 3). Next, k-means clustering (Euclidean distance) was conducted in STATISTICA with a maximum of 50 iterations and an initialization procedure aimed at maximizing the distance between cluster centroids. Three clusters differing in size were identified: Cluster 1 included 76 individuals (38%), Cluster 2 comprised 96 individuals (48%), and Cluster 3 consisted of 28 individuals (14%).

The values presented outside the diagonal of the matrix represent standardized distances between cluster centroids, whereas diagonal values equal to zero indicate no within-cluster distance. The largest inter-cluster distance was observed between Cluster 2 and Cluster 3 (d = 2.27), indicating the greatest dissimilarity in the analyzed profiles between these groups. A slightly lower, but still substantial, distance was noted between Cluster 1 and Cluster 3 (d = 2.04).

In contrast, the smallest inter-cluster distance was observed between Cluster 1 and Cluster 2 (d = 1.79), suggesting a relatively higher similarity between these two clusters. The overall pattern of inter-centroid distances supports the adequacy of the three-cluster solution, with Cluster 3 representing the most distinct subgroup within the study sample (Table 4).

**Figure 1:**
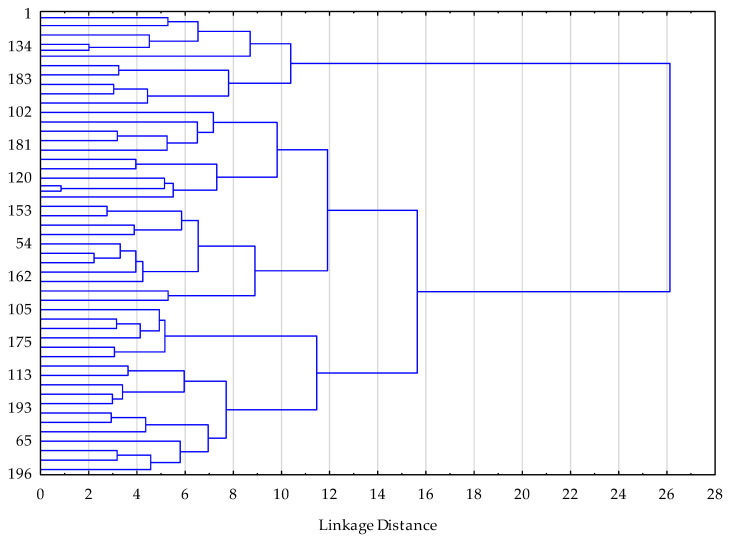
Dendrogram from hierarchical cluster analysis illustrating linkage distances between observations based on standardized psychological variables. The marked increase in linkage distance supports a three-cluster solution (k = 3).

To support the selection of a three-cluster solution (k = 3), we additionally present the dendrogram obtained from the exploratory hierarchical cluster analysis (Figure 1).

Following the identification of three distinct clusters based on the cluster analysis (Table 4, Figure 1), between-cluster comparisons were performed to examine differences in psychological and behavioral characteristics.

Significant differences between clusters were observed for trait anxiety (STAI trait; F = 10.368, η^2^ = 0.095, *p* < 0.0001) and state anxiety (STAI state; F = 4.423, η^2^ = 0.043, *p* = 0.0132). The highest levels of both anxiety dimensions were observed in Cluster 1, whereas the lowest levels were found in Cluster 2.

Significant between-cluster differences were also identified for neuroticism (F = 9.128, η^2^ = 0.085, *p* = 0.0002), with the highest scores in Cluster 1 and the lowest in Cluster 2. Extraversion also differed significantly across clusters (F = 14.187, η^2^ = 0.126, *p* < 0.0001), with the highest levels observed in Cluster 2 and the lowest in Cluster 3. Significant differences were found for openness to experience (F = 3.386, η^2^ = 0.033, *p* = 0.0358), with the highest mean values in Cluster 1 and the lowest in Cluster 2.

Among personality traits, agreeableness (F = 3.047, η^2^ = 0.030, *p* = 0.0498) and conscientiousness (F = 5.453, η^2^ = 0.053, *p* = 0.0050) also differed significantly across clusters. Cluster 2 exhibited the highest level of conscientiousness.

With respect to impulsivity, significant between-cluster differences were observed for attentional impulsivity (BIS-AI; F = 15.348, η^2^ = 0.135, *p* < 0.001), motor impulsivity (BIS-MI; F = 14.859, η^2^ = 0.131, *p* < 0.0001), non-planning impulsivity (BIS-NI; F = 5.156, η^2^ = 0.050, *p* = 0.0066), and the total BIS-11 score (F = 14.659, η^2^ = 0.130, *p* < 0.0001). The highest levels of BIS-AI, BIS-MI, and total impulsivity were observed in Cluster 1, whereas the highest BIS-NI scores were observed in Cluster 3.

In addition, significant differences between clusters were found for ADHD symptom severity (F = 14.229, η^2^ = 0.126, *p* < 0.0001), with the highest values observed in Cluster 1 and the lowest in Cluster 2. No significant between-cluster differences were observed for hedonic capacity as assessed by the SHAPS (Table 5).

Following the identification of significant between-cluster differences in psychological and behavioral characteristics (Table 5), genotype distributions were compared across the three clusters using the chi-square (χ^2^) test of independence. For each polymorphism, genotype counts within Clusters 1–3 are presented together with χ^2^ statistics and corresponding *p*-values (Table 6).

Significant between-cluster differences in genotype frequencies were observed for the majority of the analyzed polymorphisms. For *DRD2* rs1076560, a significant heterogeneity in genotype distribution was detected (χ^2^ = 58.167, *p* < 0.0001), with a predominance of the C/C genotype in Clusters 1 and 2 and a relatively higher proportion of the A/C genotype in Cluster 3.

Significant differences were also found for *DRD2* Tag1D rs1800498 (χ^2^ = 76.221, *p* < 0.0001). Cluster 1 was characterized by a predominance of the T/T genotype, whereas a more balanced distribution of T/T and C/T genotypes was observed in Cluster 2, and a higher proportion of the C/T genotype was noted in Cluster 3.

In the case of *DRD2* Tag1B rs1079597, genotype distributions differed significantly across clusters (χ^2^ = 24.364, *p* < 0.0001), with the G/G genotype predominating in all clusters, accompanied by an increased proportion of heterozygous A/G genotypes in Cluster 3 compared with Clusters 1 and 2.

Marked and significant differences between clusters were identified for *DRD2* Ex8 rs6276 (χ^2^ = 65.490, *p* < 0.0001). Cluster 1 was characterized by a predominance of the A/A genotype, Cluster 2 by the A/G genotype, whereas no single genotype predominated in Cluster 3. Similarly, genotype distributions for the *DRD2* promoter polymorphism rs1799732 differed significantly across clusters (χ^2^ = 39.541, *p* < 0.0001), with ins/ins genotypes dominating in Clusters 1 and 2 and a high proportion of ins/del genotypes observed in Cluster 3.

Significant between-cluster differences were also observed for *ANKK1* Tag1A rs1800497 (χ^2^ = 33.184, *p* < 0.0001), with Cluster 1 consisting exclusively of C/C homozygotes, whereas heterozygous C/T genotypes were present in Clusters 2 and 3. For *DAT1* (VNTR), genotype distributions differed significantly across clusters (χ^2^ = 59.654, *p* < 0.0001), with a predominance of the 9/10 genotype in Clusters 1 and 3 and the 10/10 genotype in Cluster 2. Likewise, *DRD4* Ex3 showed significant genotype distribution differences across clusters (χ^2^ = 70.541, *p* < 0.0001), with the s/l genotype predominating in Cluster 1 and the s/s genotype predominating in Clusters 2 and 3.

For genotype comparisons (Table 6), chi-square tests were applied; however, comparisons involving low expected cell counts should be interpreted cautiously due to sparse data in the smallest cluster.

**Figure 2:**
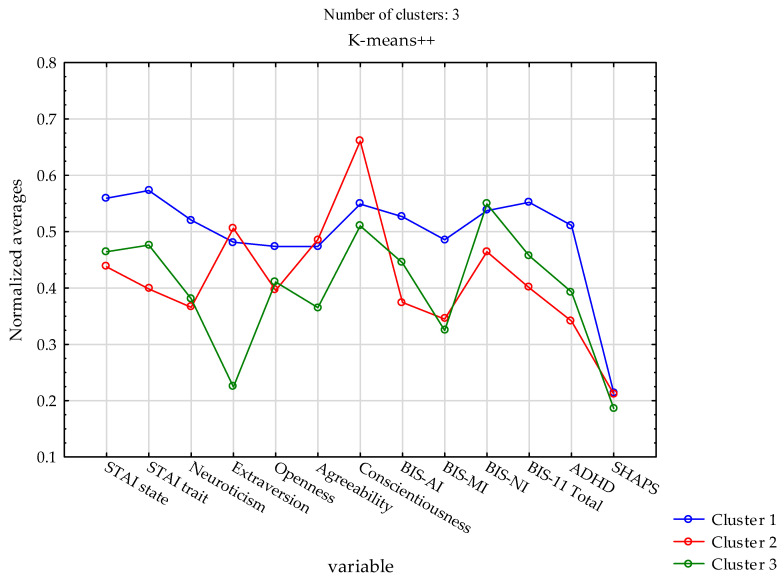
Normalized (z-score) mean profiles of personality traits, anxiety, impulsivity, ADHD symptoms, and hedonic capacity across the three clusters identified using k-means clustering analysis. Standardized scores were derived from the NEO Five-Factor Personality Inventory, State–Trait Anxiety Inventory (STAI), Barratt Impulsiveness Scale (BIS-11), Adult ADHD Self-Report Scale (ASRS v1.1), and Snaith–Hamilton Pleasure Scale (SHAPS).

The k-means cluster analysis enabled the identification of three distinct clusters characterized by different psychological, behavioral, and genetic profiles. Cluster 1 was characterized by the highest levels of trait and state anxiety, neuroticism, attentional and motor impulsivity, overall impulsivity, and ADHD symptoms, accompanied by specific distributions of *DRD2* and *DRD4* polymorphisms. Cluster 2 exhibited lower levels of anxiety and impulsivity and was distinguished by the highest levels of extraversion and conscientiousness. Cluster 3 was characterized by the lowest levels of extraversion and agreeableness, intermediate levels of impulsivity and ADHD symptoms, and the greatest genetic distinctiveness relative to the other clusters. A visual comparison of the normalized profiles across clusters is presented in Figure 2. Overall, these results indicate that the identified clusters differed not only in behavioral measures but also in the distribution of dopaminergic gene variants, supporting the distinctiveness of the derived profiles.

## 4. Discussion

The present study identified three distinct clusters characterized by differentiated psychological and genetic profiles within a cohort of elite combat sport athletes [28]. By integrating multiple behavioral dimensions with dopaminergic gene polymorphisms using a data-driven clustering approach [29], the analysis revealed heterogeneity in anxiety, impulsivity, personality traits, and ADHD symptoms that was not apparent at the level of individual variables [26]. Notably, the clusters differed not only in their psychological profiles but also in the distribution of key dopaminergic polymorphisms, supporting the relevance of combined genotype–phenotype approaches in behavioral research. In contrast, hedonic capacity did not differ significantly between clusters, suggesting that reward sensitivity, as measured in this study, may be relatively stable across distinct neurobehavioral profiles within this population. Given the cross-sectional design and the unsupervised nature of the clustering procedure, the present findings should be interpreted as exploratory and hypothesis-generating. Together, these findings suggest that multidimensional integration of behavioral and genetic data may help uncover discrete neurogenetic profiles relevant to the study of individual differences in behavior [30].

The behavioral profiles observed across the three clusters suggest distinct patterns of emotional regulation and behavioral control that are consistent with current conceptualizations in neurogenetics of behaviour. Cluster 1, characterized by elevated anxiety, impulsivity, neuroticism, and ADHD symptoms, may reflect a profile of heightened emotional and behavioral reactivity, in which regulatory demands are particularly pronounced [31]. In contrast, Cluster 2 displayed lower levels of anxiety and impulsivity alongside higher extraversion and conscientiousness, suggesting a more adaptive behavioral profile associated with effective self-regulation and emotional stability [32]. Cluster 3 occupied an intermediate position in terms of most behavioral measures but was distinguished by reduced social-oriented personality traits and pronounced genetic distinctiveness, suggesting that similar behavioral outcomes may arise from different underlying genetic configurations [33]. Taken together, these patterns underscore the multidimensional nature of behavioral regulation and highlight that neurobehavioral phenotypes are best understood as integrated constellations of traits rather than isolated dimensions [34,35].

The observed differences in genotype distributions across clusters are compatible with a role of dopaminergic signaling pathways in behavioral profiles, while simultaneously underscoring the limitations of single-gene interpretations [36]. Polymorphisms within genes such as *DRD2*, *ANKK1*, *DAT1*, and *DRD4* have previously been implicated in individual differences in emotional reactivity, impulsivity, attentional control, and reward-related behaviors [37]; however, the present findings suggest that their effects may be expressed at the level of integrated behavioral constellations rather than isolated traits [38]. Importantly, no single polymorphism uniquely defined any cluster, suggesting that the behavioral phenotypes identified here likely arise from combined and context-dependent genetic effects [39]. This pattern is consistent with contemporary neurogenetic models emphasizing polygenic contributions and complex genotype–phenotype relationships, in which genetic variation modulates vulnerability and regulation across multiple behavioral domains rather than exerting deterministic effects on specific outcomes [40].

The use of an unsupervised cluster-based approach represents a key methodological strength of the present study, as it allows for the identification of naturally occurring neurobehavioral profiles without imposing predefined assumptions regarding trait boundaries or genetic effects [41]. Traditional association analyses often focus on single behavioral dimensions or individual genetic variants, which may obscure meaningful patterns arising from the interaction of multiple traits and genes [18]. By integrating standardized psychological measures into a multidimensional framework, the clustering approach provides a complementary framework for characterizing behavioral heterogeneity and reflects the complexity of real-world behavioral regulation [42,43]. This integrative strategy aligns with current trends in neurogenetics of behaviour, which increasingly emphasize data-driven methods to disentangle complex genotype–phenotype relationships and to move beyond reductionist interpretations of behavioral variability [44].

Elite combat sport athletes constitute a particularly informative model for investigating neurogenetic mechanisms underlying behavioral regulation, as they are exposed to sustained cognitive, emotional, and physical demands that require precise control of anxiety, impulsivity, attention, and motivation. The high level of selection and long-term training characteristic of this population may amplify individual differences in regulatory traits, thereby facilitating the identification of distinct neurobehavioral profiles [26]. At the same time, these features necessitate caution when generalizing the present findings beyond athletic contexts, as the observed profiles likely reflect an interaction between genetic predispositions and prolonged adaptive processes specific to high-performance environments [45]. Therefore, the identified profiles likely reflect the combined contribution of genetic predispositions and prolonged high-performance sport exposure. Nevertheless, studying such highly regulated populations offers valuable insights into how genetic variability manifests under conditions of repeated behavioral challenge, contributing to a deeper understanding of neurogenetic influences on behavior [46]. Previous studies conducted in non-athletic and mixed-gender samples have also reported mixed associations between dopaminergic polymorphisms and traits such as impulsivity, anxiety, and attentional control [9,12]; however, effect sizes and directions are often heterogeneous and context-dependent [39,40]. This underscores that the present cluster structure and genotype distributions may reflect sport-specific selection and long-term training-related adaptation, and should be interpreted cautiously when extrapolating beyond elite combat sport populations.

Several limitations of the present study should be acknowledged. First, the study sample consisted exclusively of male athletes, which limits the generalizability of the findings to female populations and to non-athletic groups. Second, the cross-sectional design precludes conclusions regarding causal relationships between genetic variation and behavioral profiles, as well as the assessment of developmental or training-related changes over time. Moreover, variables such as training volume, years of training, recent competition exposure, length of athletic career, and acute psychological stress were not systematically assessed and therefore could not be included as covariates, which may have introduced residual confounding. Although correlations among some psychological dimensions were expected due to construct overlap, multicollinearity may have influenced the structure of the derived clusters. Additionally, Cluster 3 was relatively small (*n* = 28), which may reduce statistical power for genotype distribution analyses and increase uncertainty of effect estimates, particularly for low-frequency genotypes; therefore, these findings should be interpreted cautiously and validated in larger samples. Third, although the study focused on well-established candidate genes within the dopaminergic system, it did not include genome-wide analyses or functional biological measures such as neuroimaging, neurochemical markers, or epigenetic modifications, which could provide deeper insight into underlying mechanisms. Finally, behavioral traits were assessed using self-report instruments, which, while validated and widely used, may be influenced by response biases. These limitations highlight the need for cautious interpretation of the results and underscore the importance of complementary approaches in future research.

Collectively, the present findings underscore the value of integrative, data-driven approaches in advancing the neurogenetics of behaviour. By simultaneously considering multiple behavioral dimensions and genetic variants, the study demonstrates that meaningful neurobehavioral patterns emerge at the level of profiles rather than isolated traits or single polymorphisms. This perspective reinforces the view that behavioral regulation is best understood as a multidimensional construct shaped by complex interactions among genetic, psychological, and contextual factors.

Building on this integrative framework, future research should aim to extend cluster-based neurogenetic analyses to more diverse populations, including female participants and non-athletic cohorts, to assess the generalizability of the identified profiles. Longitudinal designs could further elucidate how neurogenetic profiles evolve over time in response to environmental demands and training-related adaptations. Moreover, the incorporation of additional biological layers, such as epigenetic markers, neuroimaging data, and genome-wide approaches, may help to clarify the mechanisms linking genetic variation to behavioral regulation [47]. Together, these directions hold promise for refining our understanding of genotype–phenotype relationships and for advancing a more comprehensive, systems-level view of behavioral diversity within the neurogenetics of behaviour framework.

## 5. Conclusions

The present study demonstrates that integrating multidimensional psychological assessments with dopaminergic genetic variation enables the identification of distinct neurogenetic profiles within a highly selected population of elite combat sport athletes. Using a cluster-based, data-driven approach, three differentiated profiles were identified, characterized by unique constellations of anxiety, impulsivity, personality traits, ADHD symptoms, and genotype distributions. These findings highlight that behavioral regulation is best understood at the level of integrated profiles rather than isolated traits or single genetic variants.

Importantly, the results support the relevance of dopaminergic polymorphisms in shaping complex behavioral phenotypes while underscoring the polygenic and context-dependent nature of genotype–phenotype relationships. The absence of between-cluster differences in hedonic capacity further suggests that certain behavioral dimensions may remain relatively stable across distinct neurobehavioral configurations within this population.

Overall, this study advances the neurogenetics of behaviour framework by illustrating the value of cluster-based methodologies for capturing behavioral heterogeneity and linking it to genetic variability. The proposed integrative approach provides a foundation for future research aimed at refining behavioral phenotyping and exploring neurogenetic mechanisms across diverse populations and environmental contexts.

## Figures and Tables

**Table 1:** Descriptive characteristics of personality traits, anxiety, impulsivity, ADHD symptoms, and hedonic capacity in the study sample assessed using the NEO Five-Factor Personality Inventory, State–Trait Anxiety Inventory (STAI), Barratt Impulsiveness Scale (BIS-11), Adult ADHD Self-Report Scale (ASRS v1.1), and Snaith–Hamilton Pleasure Scale (SHAPS).

		95% CI				
Variable	*M*	LL	UL	*Mdn*	*SD*	*n*
Age (years)	22.86	21.99	23.73	21.00	6.25	200
STAI state	4.90	4.59	5.21	5.00	2.20	200
STAI trait	5.28	4.95	5.61	5.00	2.36	200
Neuroticism	4.84	4.52	5.16	5.00	2.28	200
Extraversion	6.20	5.94	6.46	6.00	1.87	200
Openness	4.42	4.20	4.64	4.00	1.61	200
Agreeability	5.18	4.89	5.47	5.00	2.11	200
Conscientiousness	7.18	6.91	7.45	7.00	1.91	200
BIS-AI	15.95	15.47	16.43	16.00	3.44	200
BIS-MI	21.70	21.10	22.30	21.00	4.32	200
BIS-NI	24.58	24.12	25.03	24.00	3.26	200
BIS-11 Total	61.97	60.75	63.19	62.00	8.76	200
ADHD	22.48	20.87	24.09	23.50	11.53	200
SHAPS	46.43	45.29	47.58	45.00	8.19	200

*M*—mean; CI—confidence interval; LL—lower limit of the 95% confidence interval; UL—upper limit of the 95% confidence interval; *Mdn*—median; *SD*—standard deviation; *n*—number of subjects.

**Table 2:** Pearson correlation heatmap of psychological traits. Positive correlation is marked in blue, negative correlation in red. Correlation |r| > 0.8 indicates collinearity between variables ǂ.

	STAI Trait	Neuroticism	Extraversion	Openness	Agreeability	Conscientiousness	BIS-AI	BIS-MI	BIS-NI	BIS-11 Total	ADHD	SHAPS
STAI ST Sten	0.828 ǂ	0.686	−0.303	0.052	−0.286	−0.283	0.525	0.551	0.250	0.559	0.548	0.184
STAI trait		0.790	−0.442	0.075	−0.346	−0.521	0.582	0.583	0.301	0.602	0.633	0.129
Neuroticism			−0.275	0.244	−0.299	−0.506	0.544	0.466	0.360	0.548	0.555	−0.027
Extraversion				0.159	0.256	0.322	−0.144	−0.062	−0.110	−0.084	−0.098	0.123
Openness					−0.117	−0.123	−0.085	0.102	−0.001	0.024	−0.007	0.080
Agreeability						0.356	−0.374	−0.409	0.065	−0.356	−0.4661	0.040
Conscientiousness							−0.462	−0.417	−0.463	−0.536	−0.331	−0.111
BIS-AI								0.717	0.415	0.886	0.633	0.083
BIS-MI									0.295	0.873	0.595	0.141
BIS-NI										0.616	0.266	−0.0531
BIS-11 Total											0.627	0.098
ADHD												−0.008

**Table 3:** Genotype frequencies of *DRD2* (rs1076560, Tag1D rs1800498, Tag1B rs1079597, Ex8 rs6276, promoter rs1799732), *ANKK1* (Tag1A rs1800497), *DAT1* (VNTR), and *DRD4* (Ex3 VNTR) polymorphisms in the study sample.

Variable	Homozygous Wild *n* (%)	Heterozygous *n* (%)	Homozygous Recessive *n* (%)
*DRD2* rs1076560	C/C	A/C	A/A
	160 (80%)	32 (16%)	8 (4%)
*DRD2* Tag1D rs1800498	T/T	C/T	C/C
	120 (60%)	56 (28%)	24 (12%)
*DRD2* Tag1B rs1079597	G/G	A/G	A/A
	172 (86%)	28 (14%)	0 (0%)
*DRD2* Ex8 rs6276	A/A	A/G	G/G
	84 (42%)	76 (38%)	40 (20%)
*DRD2* promoter rs1799732	ins/ins	ins/del	del/del
	148 (74%)	52 (26%)	0 (0%)
*ANKK1* Tag1A rs1800497	C/C	C/T	T/T
	160 (80%)	40 (20%)	0 (0%)
*DAT1* (VNTR)	10/10	9/10	9/9
	96 (48%)	96 (48%)	8 (4%)
*DRD4* Ex3	s/s	s/l	l/l
	116 (58%)	80 (40%)	4 (2%)

*n*—number of subjects, VNTR—variable number tandem repeat.

**Table 4:** Cluster sizes and standardized distances between cluster centroids obtained from k-means clustering.

	Cluster 1 *n* = 76 (38%)	Cluster 2 *n* = 96 (48%)	Cluster 3 *n* = 28 (14%)
Cluster 1	0.00	1.79	2.04
Cluster 2	1.79	0.00	2.27
Cluster 3	2.04	2.27	0.00

*n*—number of subjects; Values represent standardized Euclidean distances between cluster centroids.

**Table 5:** Comparison of the three identified clusters in terms of personality traits, anxiety, impulsivity, ADHD symptoms, and hedonic capacity.

Variable	Cluster 1 (*n* = 76) *M* ± *SD*	Cluster 2 (*n* = 96) *M* ± *SD*	Cluster 3 (*n* = 28) *M* ± *SD*	F	η^2^	*p*
STAI state	5.47 ± 2.29	4.50 ± 1.86	4.71 ± 2.71	4.423	0.043	0.0132 *
STAI trait	6.16 ± 2.32	4.58 ± 2.11	5.29 ± 2.54	10.368	0.095	<0.0001 *#
Neuroticism	5.68 ± 2.22	4.29 ± 2.00	4.43 ± 2.71	9.128	0.085	0.0002 *#
Extraversion	6.36 ± 2.04	6.54 ± 1.69	4.57 ± 0.92	14.187	0.126	<0.0001 *#
Openness	4.78 ± 1.97	4.17 ± 1.31	4.28 ± 1.18	3.386	0.033	0.0358 *
Agreeability	5.26 ± 2.31	5.37 ± 1.88	4.28 ± 2.16	3.047	0.030	0.0498 *
Conscientiousness	6.84 ± 1.91	7.62 ± 1.58	6.57 ± 2.54	5.453	0.053	0.0050 *
BIS-AI	17.47 ± 3.52	14.74 ± 2.96	16.00 ± 3.17	15.348	0.135	<0.0001 *#
BIS-MI	23.68 ± 5.44	20.58 ± 2.55	20.14 ± 3.83	14.859	0.131	<0.0001 *#
BIS-NI	25.21 ± 3.45	23.82 ± 2.92	25.43 ± 3.39	5.156	0.050	0.0066 *
BIS-11 Total	65.84 ± 9.87	59.02 ± 6.53	61.57 ± 8.48	14.659	0.130	<0.0001 *#
ADHD	27.58 ± 11.71	18.75 ± 10.49	21.43 ± 9.38	14.229	0.126	<0.0001 *#
SHAPS	46.78 ± 12.00	46.62 ± 5.07	44.86 ± 1.38	0.608	0.006	0.5454

*n*—number of subjects, *M*—mean; *SD*—standard deviation; *—statistically significant difference (*p* < 0.05); F—ANOVA F statistic. # Bonferroni correction, α = 0.0038 (0.05/13). Effect size (η^2^) was interpreted following Cohen’s conventions: 0.01 = small, 0.06 = medium, 0.14 = large.

**Table 6:** Genotype distribution differences across clusters for *DRD2*, *ANKK1*, *DAT1*, and *DRD4* polymorphisms.

Variable	Cluster 1 *n* = 76			Cluster 2 *n* = 96			Cluster 3 *n* = 28			χ^2^	*p*
*DRD2* rs1076560	C/C	A/C	A/A	C/C	A/C	A/A	C/C	A/C	A/A	58.167	<0.0001 *#
	68	4	4	84	12	0	8	16	4		
*DRD2* Tag1D rs1800498	T/T	C/T	C/C	T/T	C/T	C/C	T/T	C/T	C/C	76.221	<0.0001 *#
	72	0	4	44	36	16	4	20	4		
*DRD2* Tag1B rs1079597	G/G	A/G	A/A	G/G	A/G	A/A	G/G	A/G	A/A	24.364	<0.0001 *#
	72	4	0	84	12	0	16	12	0		
*DRD2* Ex8 rs6276	A/A	A/G	G/G	A/A	A/G	G/G	A/A	A/G	G/G	65.490	<0.0001 *#
	56	8	12	16	60	20	12	8	8		
*DRD2* promoter rs1799732	ins/ins	ins/del	del/del	ins/ins	ins/del	del/del	ins/ins	ins/del	del/del	39.541	<0.0001 *#
	68	8	0	72	24	0	8	20	0		
*ANKK1* Tag1A rs1800497	C/C	C/T	T/T	C/C	C/T	T/T	C/C	C/T	T/T	33.184	<0.0001 *#
	76	0	0	68	28	0	16	12	0		
*DAT1*	10/10	9/10	9/9	10/10	9/10	9/9	10/10	9/10	9/9	59.654	<0.0001 *#
	24	52	0	68	20	8	4	24	0		
*DRD4* Ex3	s/s	s/l	l/l	s/s	s/l	l/l	s/s	s/l	l/l	70.541	<0.0001 *#
	16	56	4	76	20	0	24	4	0		

*n*—number of subjects; χ^2^—the chi-square test; *p*—*p*-value; *—statistically significant difference (*p* < 0.05), l—long allele; s—short allele. # Bonferroni correction, α = 0.00625 (0.05/8).

## Data Availability

The data presented in this study are available on request from the corresponding author. The data are not publicly available due to privacy concerns.
